# Liquid Superlubricity Enabled by the Synergy Effect of Graphene Oxide and Lithium Salts

**DOI:** 10.3390/ma15103546

**Published:** 2022-05-16

**Authors:** Xiangyu Ge, Zhiyuan Chai, Qiuyu Shi, Yanfei Liu, Jiawei Tang, Wenzhong Wang

**Affiliations:** 1 School of Mechanical Engineering, Beijing Institute of Technology, Beijing 100081, China; gexy@bit.edu.cn (X.G.); chaizycn@163.com (Z.C.); tjw1578840479@163.com (J.T.); wangwzhong@bit.edu.cn (W.W.); 2 State Grid Smart Grid Research Institute Co., Ltd., Beijing 102209, China; shiqiuyu@geiri.sgcc.com.cn

**Keywords:** superlubricity, graphene oxide, LiPF_6_, friction reduction

## Abstract

In this study, graphene oxide (GO) nanoflakes and lithium salt (LiPF_6_) were utilized as lubrication additives in ether bond−containing dihydric alcohol aqueous solutions (DA(aq)) to improve lubrication performances. The apparent friction reduction and superlubricity were realized at the Si_3_N_4_/sapphire interface. The conditions and laws for superlubricity realization have been concluded. The underlying mechanism was the synergy effect of GO and LiPF_6_. It was proven that a GO adsorption layer was formed at the interface, which caused the shearing interface to transfer from solid asperities to GO interlayers (weak interlayer interactions), resulting in friction reduction and superlubricity realization. In addition to the GO adsorption layer, a boundary layer containing phosphates and fluorides was formed by tribochemical reactions of LiPF_6_ and was conducive to low friction. Additionally, a fluid layer contributed to friction reduction as well. This work proved that GO−family materials are promising for friction reduction, and provided new insights into realizing liquid superlubricity at macroscale by combining GO with other materials.

## 1. Introduction

According to statistics, the annual economic losses of energy consumption, material loss, and equipment maintenance caused by friction and wear in most industrial countries account for 2–7% of their GDP [1,2]. Therefore, it is urgent to develop efficient lubricating materials to reduce friction and wear and thus reduce economic losses. The concept of superlubricity was put forward by Hirano and Shinjo in the early 1990s [3], which specifically refers to the lubrication state when the sliding coefficient of friction (COF) is at 0.001 magnitude or even lower [4]. Because of the minimal friction resistance, superlubricity can greatly reduce friction−induced energy loss. Superlubricity is mainly divided into solid superlubricity and liquid superlubricity according to the materials. Solid materials that achieve superlubricity are mostly two−dimensional (2D) materials with a layered structure, such as molybdenum disulfide [5], graphite [6], diamond−like film, and carbon and nitrogen film [7]. The solid superlubricity mechanisms are mainly attributed to surface incommensurate contact, weak interaction, and Coulomb repulsion [8]. In terms of liquid superlubricity, it has been proven that this can be achieved by ceramic water lubrication [9], phosphoric acid aqueous solution [10], salt [11] and ionic liquid solution [12], biological mucus [13], polymer molecular brush [14], polyol solution [15], polyether solution [16], and poly−α olefin with boron nitride [17], etc.

Graphene oxide (GO), as a prevalent 2D material, presents superb properties in various domains and exhibits superb tribological properties as additives in liquid lubrication as well, such as friction reduction and antiwear properties [18]. In recent years, our group has realized macroscale liquid superlubricity by utilizing GO nanoflakes as additives in alcohol aqueous solution [19], and by merging GO with a kind of “in−situ” formed ionic liquid, the contact pressure during the superlubricity period was extended to a high contact pressure of 600 MPa [20]. Afterward, GO nanoflakes were used as additives to realize superlubricity on DLC film [21]. These researches proved that GO has substantial potential for realizing liquid superlubricity at the macroscale. However, the base liquid for GO that achieved superlubricity was limited to typical alcohols (that do not contain other chemical bonds). Whether and how can GO facilitate the realization of superlubricity in other kinds of liquids is yet to be revealed. Besides, lithium salts such as LiPF_6_ have been well documented to show excellent friction reduction and wear resistance properties in ether bond−containing alcohols [2]. Therefore, LiPF_6_ was chosen as an additive and combined with GO to achieve an improvement in lubrication performance. This work used dihydric alcohol (DA) that contains an ether bond as the base liquid, and friction reduction was realized when both GO and lithium salt (LiPF_6_) were used as additives. Especially when DA viscosity was suitable, superlubricity was realized. The fundamental mechanisms of friction reduction and superlubricity for GO and LiPF_6_ are discussed herein.

## 2. Materials and Methods

### 2.1. Materials

**Figure 1:**
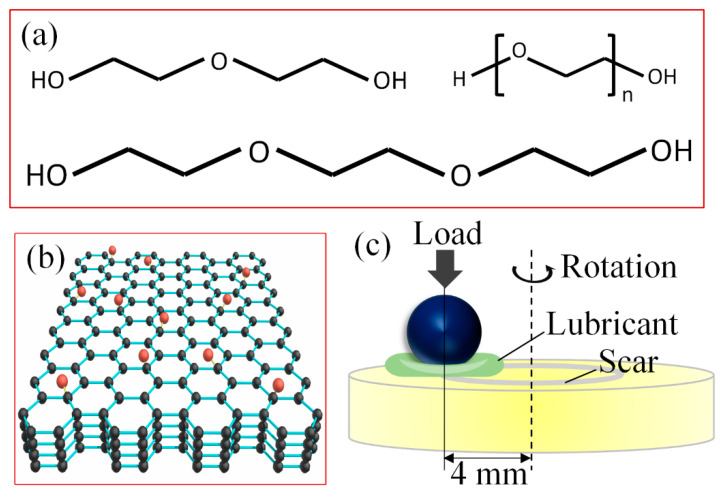
(**a**) The structure of DAs, (**b**) layered structure of graphene oxide, and (**c**) diagram of testing model.

The DAs with ether bonds (Figure 1a) have a purity of over 99%, including diethylene glycol (DEG), triethylene glycol (TEG), and polyethylene glycol (PEG) with an average molecular weight of 200 g/mol. GO nanoflakes (Figure 1b) were provided by the Aladdin Industrial Corporation. The lithium salt LiPF_6_ has a purity of over 97%. All these reagents were offered by the Aladdin Industrial Corporation and were used without further treatment. DA(aq) was formulated by blending alcohol with deionized water with the ratio of 1:5 by weight and was referred to as DEG(aq), TEG(aq), and PEG(aq). The GO containing DA(aq) was formulated by blending GO with DA(aq) with a ratio of 1:600 by weight through sonication. The LiPF_6_ containing DA(aq) was formulated by dissolving LiPF_6_ in DA(aq) with a ratio of 1:120 by weight. The GO and LiPF_6_ containing DA(aq) was formulated by adding both GO and LiPF_6_ into DA(aq) with the above ratio.

The viscosities of alcohols at room temperature were evaluated by a Rheometer (MCR101, Anton Paar, Austria). The layered structure of GO nanoflakes was evaluated by a high−resolution transmission electron microscope (HRTEM; 2100F, JEM, Japan). To obtain the morphology of GO nanoflakes, 15 μL GO(aq) was added to mica substrate and dried in a vacuum oven for 2 h to remove moisture. Subsequently, an atomic force microscope (AFM, Icon, Bruker, Germany) was used for microscopic observation of the dried GO nanoflakes. The lateral diameter distribution of GO nanoflakes was evaluated by a Zetasizer (Nano−Zs, Malvern) in an aqueous solution. The C/O ratio and chemical bonds of GO were determined by an X−ray photoelectron spectroscope (XPS, PHI QUANTERA−II SXM, ULVAC−PHI, Japan). A Raman spectrometer (LabRAM HR Evolution, HORIBA Jobin Yvon, Japan) was used to characterize the chemical groups, vibration features, and defects in GO.

### 2.2. Tribological Experiments

A universal micro−tribometer (UMT−3, Bruker) was used to conduct the friction test under a ball on disk rotation condition (Figure 1c). The friction pair consisted of a silicon nitride ball (Si_3_N_4_, Ø4 mm, *Ra* ≈ 10 nm) and a sapphire disk (*Ra* ≈ 1 nm). Before testing, the ball and disk were cleaned by ultrasonic with acetone and ethanol for 15 min, then washed with deionized water, and dried with compressed air. During testing, 20 μL solution was dropped to the contact area of the friction pair. The load range was 2–4 N and the Hertzian contact pressure was approximately 1.5–1.9 GPa. The sliding speed was 12.5–250 mm/s. Each test was repeated three times and the measurement accuracy of the friction coefficient was ±0.001. To reduce the measurement error as much as possible, the levelness of the loading platform and the verticality of the loading device were adjusted so that the same COF value can be obtained while testing under clockwise and counterclockwise rotation. All tests were carried out at room temperature with a relative humidity of 10–30%.

### 2.3. Surface Characterization

After the friction test, the worn scar diameter (WSD) and roughness of worn surface on the ball and disk were measured by an optical microscope and a white−light interferometer (Nexview, ZYGO Lamda, USA). A scanning electron microscope (SEM, S4800, Hitachi, Japan) was used to observe the topographies of worn surfaces on the ball and disk. The worn surfaces were coated with a very thin platinum coating to increase the image contrast before SEM examination. To study the chemical features of the tribolayer formed during friction testing, XPS and Raman spectrometry were used to characterize the presence of chemical elements and chemical groups on the worn surfaces. HRTEM samples were prepared by a focused ion beam (FIB, LYRA3, Tescan, Czech Republic) to obtain images of cross−sectional areas in the worn area. The chosen sections from the worn area were 10 μm in length. A Cr layer (20 nm thick) was sputtered onto the worn surfaces. Then, a Pt protection layer (100 nm thick) was deposited before performing FIB.

## 3. Results

### 3.1. Material Characterization

**Figure 2:**
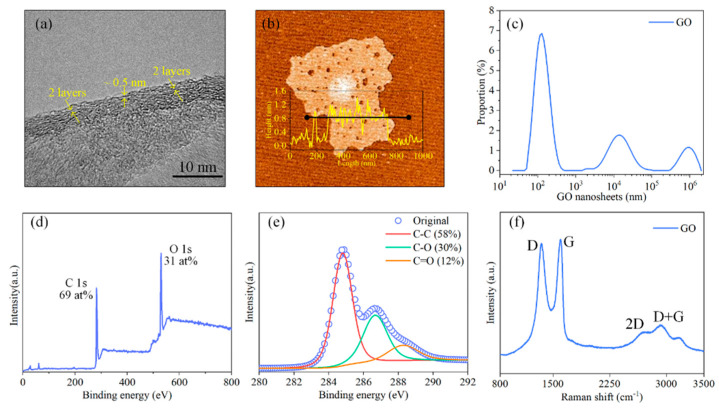
(**a**) HRTEM image showing the layered structure of GO nanoflakes with an interlayer spacing of approximately 0.5 nm, (**b**) AFM image showing the thickness of GO single layer is approximately 0.8 nm, (**c**) lateral size distribution of GO nanoflakes, (**d**) XPS spectrum of GO nanoflakes showing the C/O ratio is approximately 69:31, (**e**) XPS spectrum showing chemical bond within GO, and (**f**) Raman spectrum showing the typical D, G, 2D, and D + G bands of GO.

The layered structure of GO nanoflakes was observed via HRTEM as shown in Figure 2a, the interlayer spacing was approximately 0.5 nm. The thickness of a single layer was approximately 0.8 nm according to AFM observation, as shown in Figure 2b. The lateral size distribution results showed that most GO nanoflakes were distributed between 100 and 200 nm, a few of them were distributed at 10 μm, and a very small amount of them were distributed at 1 mm (Figure 2c). The atom concentrations of carbon and oxygen in GO nanoflakes were approximately 69 at% and 31 at%, respectively, as shown in the XPS spectrum of GO nanoflakes (Figure 2d). Moreover, the C 1s peaks of GO nanoflakes were deconvoluted into three chemically shifted parts. The peak detected at 284.8 eV in nanoflakes was assigned to the non−oxygen carbon of C–H and C–C bonds [22]. The peak detected at 286.6 eV was assigned to C–O bonds [23]. The peak detected at 288.1 eV was assigned to C=O bonds [19] (Figure 2e). The vibration and defect characteristics of GO nanoflakes were detected by a Raman spectroscope. As shown in Figure 2f, the peak around 1350 cm^−1^ was the typical D band of GO nanoflakes, which represented the distortions, vacancies, and defects within GO. The peak around 1590 cm^−1^ was the typical G band, which represented the stretching of C–C bonds within GO [24,25]. Besides, the typical 2D and D + G bands around 2670 cm^−1^ and 2930 cm^−1^ were observed, respectively [26,27].

### 3.2. Tribological Experiments

**Figure 3:**
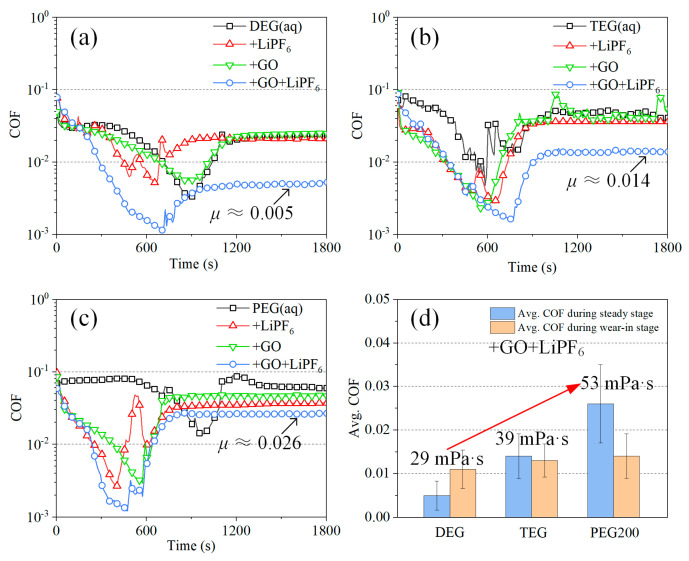
COFs of various DA(aq) acquired at 3 N, 100 mm/s, and 20 μL, (**a**) DEG(aq), (**b**) TEG(aq), (**c**) PEG(aq), and (**d**) effect of DA viscosities on COFs during wear−in and steady lubrication stages.

The friction test results are shown in Figure 3. The lubrication process of all solutions shared the same evolution trend, which contained a wear−in stage and a steady lubrication stage. The COFs were reduced rapidly during the wear−in stage and finally reached a steady state. The COF of DEG(aq) during steady lubrication stage was stable at approximately 0.022. With the addition of LiPF_6_ and GO, the COF was stable at 0.022 and 0.024 with little change, respectively. However, when LiPF_6_ and GO were introduced simultaneously, the COF of DEG(aq) + GO + LiPF_6_ was stabilized at approximately 0.005, indicative of superlubricity achievement. The same phenomenon was found when TEG(aq) and PEG(aq) were used as base liquid, as shown in Figure 3b,d. There was no significant friction reduction with the addition of LiPF_6_ or GO, and apparent friction reduction was observed when LiPF_6_ and GO were introduced simultaneously. Notably, the reduction amplitude in friction became smaller with the growth in DA viscosity. It was also found that when both LiPF_6_ and GO were introduced, the average COF was increased with the growth in DA viscosity at both the wear−in and steady lubrication states. The lowest COF and superlubricity were achieved when DEG was used as base liquid. These results clearly showed that the synergy effect of GO and LiPF_6_ contributed to the friction reduction and superlubricity achievement. It was also implied that a fluid layer functioned during the steady lubrication stage, and the influence of fluid layer increased with the growth in DA viscosity.

**Figure 4:**
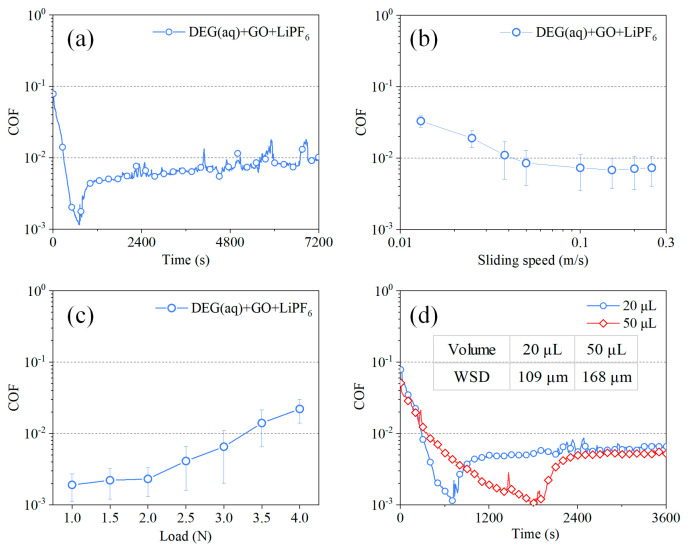
COFs of GO and LiPF_6_ containing DEG(aq) under various conditions, (**a**) long run (3 N, 100 mm/s, and 20 μL), (**b**) sliding speed (3 N and 20 μL), (**c**) normal load (100 mm/s and 20 μL), and (**d**) solution volume (3 N and 100 mm/s).

A long−run test was conducted on DEG(aq) + GO + LiPF_6_. As shown in Figure 4a, the COF remained stable at superlubricity state for nearly 2 h after a wear−in stage of 700 s. The influence of sliding velocity on COF during the superlubricity stage is shown in Figure 4b. When the sliding velocity was varied in the range of 0.05–0.25 m/s, the corresponding COFs were all lower than 0.01, demonstrating the achievement of superlubricity. When the sliding velocity was slower than 0.05 m/s, the COFs grew to larger than 0.01, demonstrating the failure of superlubricity. Therefore, there was a suitable velocity range (0.05–0.25 m/s) for superlubricity achievement with the lubrication of DEG(aq) + GO + LiPF_6_. The influence of normal loads on COF was studied with the lubrication of DEG(aq) + GO + LiPF_6_ (Figure 4c). When the load increased from 1 to 3 N, although the corresponding COF increased from 0.002 to 0.008, they were all still in the superlubricity level (COF < 0.01). When the load further increased to 3.5 N, the corresponding COF reached 0.015, demonstrating the failure of superlubricity. The influence of solution volume on the average COFs during superlubricity stage was studied (Figure 4d). When the solution volume increased from 20 µL to 50 µL, the time of wear−in stage increased significantly from approximately 700 s to 1800 s, and the WSDs increased from 109 μm to 168 μm. However, the change in solution volume affected the COF during the superlubricity stage slightly. These results showed that DEG(aq) + GO + LiPF_6_ could realize robust liquid superlubricity, and there was an optimal speed and load range for superlubricity achievement. Besides, the solution volume only affected the duration time of wear−in stage; it had little influence on the COF during the superlubricity stage.

## 4. Discussion

**Figure 5:**
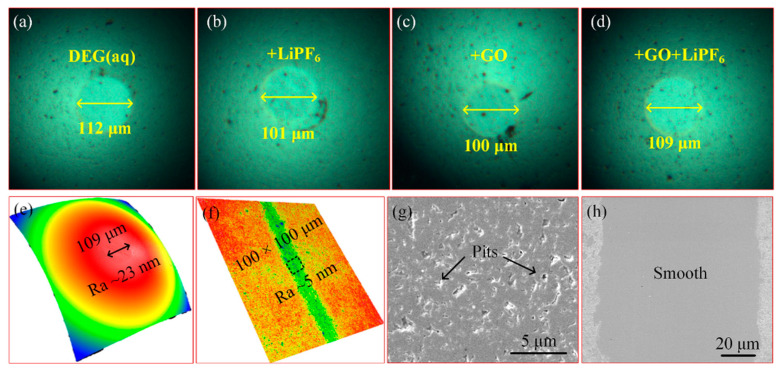
(**a**–**d**) WSDs of the balls: (**a**) DEG(aq), (**b**) +LiPF_6_, (**c**) +GO, and (**d**) +GO + LiPF_6_; (**e**,**f**) worn area *Ra* lubricated with DEG(aq) + GO + LiPF_6_ obtained by a white−light interferometer: (**e**) ball and (**f**) disk; (**g**,**h**) topographies of worn areas lubricated with DEG(aq) + GO + LiPF_6_ obtained by SEM: (**g**) ball and (**h**) disk. The worn area was obtained under the condition of 3 N, 100 mm/s, and 20 μL.

To reveal the fundamental mechanisms associated with the good friction reduction capacity and superlubricity achieved using DEG(aq) + GO + LiPF_6_, the WSDs, worn surface roughness, and topographies of worn areas were explored (Figure 5). All WSDs of balls were similar (approximately 100–110 μm), indicating the contact area, and thus the contact pressures were similar among solutions. The worn area of the ball lubricated with DEG(aq) + GO + LiPF_6_ contained many pits and thus was relatively rough (*Ra* ≈ 23 nm), whereas the worn area of the disk was relatively smooth with a roughness of approximately 5 nm. As abovementioned, a fluid layer may have existed at the interface. To study the lubrication regime during superlubricity stage with the lubrication of DEG(aq) + GO + LiPF_6_, the Hamrock–Dowson (H−D) formula was used to estimate the thickness (*h*) of the lubrication layer at the interface as detailed in our previous works [19,20]. The result showed that the fluid layer thickness was approximately 29.5 nm during the superlubricity stage. The ratio of the fluid layer thickness to the equivalent *Ra* value of two worn areas was employed to ensure the lubrication regime, which was calculated using the equation $\lambda =\frac{h}{\sqrt{\sigma_{1}^{2}+\sigma_{2}^{2}}}$ (*σ*_1_ and *σ*_2_ were the surface roughness of worn surfaces), and *λ* was calculated as 1.25. This result indicated that the lubrication regime was the mixed lubrication regime during the superlubricity stage.

**Figure 6:**
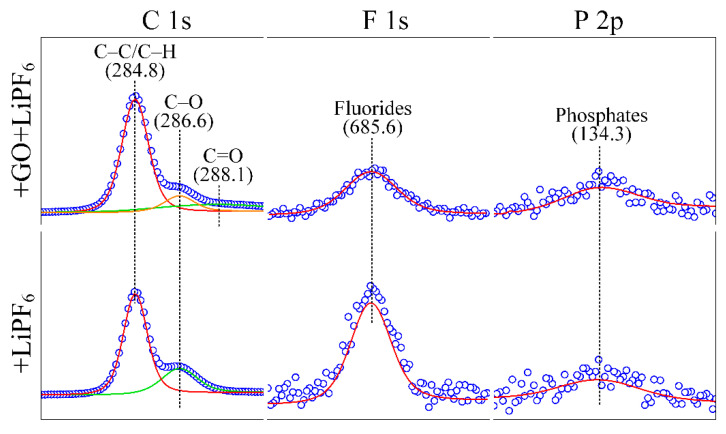
Chemical state of worn areas on Si_3_N_4_ balls with the lubrication of various solutions.

**Figure 7:**
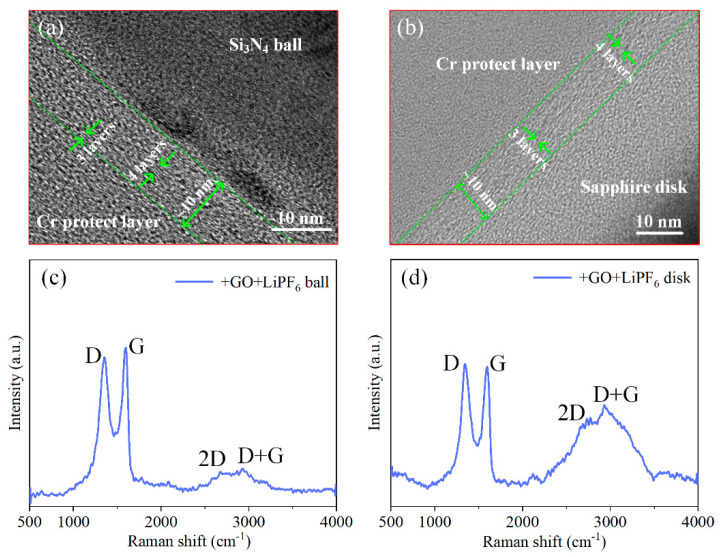
(**a**,**b**) HRTEM images of GO adsorption layer on worn surfaces. (**c**,**d**) Raman spectra of GO adsorption layer on worn areas.

To analyze the probable friction reduction and superlubricity mechanisms, the chemical state of matters on worn areas of the balls lubricated with DEG(aq) + GO + LiPF_6_ and DEG(aq) + LiPF_6_ was studied (Figure 6). On both worn areas, the peaks (284.8 and 286.6 eV) represented the carbon in aliphatic chains (C−C and C−H) and C−O bonds were detected [22,23], which may have stemmed from DEG or GO. Because there were no C=O bonds in DEG, the C=O bonds (288.1 eV) [19] detected only on the worn area with the lubrication of DEG(aq) + GO + LiPF_6_ implied the adsorption of GO nanoflakes. This result indicated that GO may have been adsorbed on the worn area to form an adsorption layer of GO, and the extremely low shear stress of GO interlayers contributed to friction reduction. The elements that stemmed from LiPF_6_ (F 1s and P 2p) were detected as well on both worn areas, which were in the state of fluorides (685.6 eV) and phosphates (134.3 eV) [28,29]. These results are consistent with our previous work, in which anions (PF_6_^−^) were transformed into fluorides and phosphates during the wear−in stage; and phosphates and fluorides had good lubrication properties [12,20,30], and thus contributed to the friction reduction. Moreover, it has been well documented that a SiO_2_−containing layer was formed at the interface when Si_3_N_4_ was lubricated with aqueous solutions. The classic tribochemical reaction was Si_3_N_4_ + 6 H_2_O → 3 SiO_2_ + 4 NH_3_ [31]. These results indicated that a tribochemical layer and a GO adsorption layer were formed at the interface and contributed to the friction reduction and superlubricity achievement. To ensure that there were GO nanoflakes adsorbed on worn areas, HRTEM and Raman observation were utilized. The GO adsorption layer on worn surfaces was directly observed by the HRTEM (Figure 7a,b). The thickness of adsorption layers was approximately 10 nm. The results shown in Figure 7c,d further confirm the adsorption of GO nanoflakes on both worn areas of the friction pair, presenting the typical D band (1350 cm^−1^), G band (1595 cm^−1^), 2D band (2700 cm^−1^), and D + G band (2935 cm^−1^) [24,25,26,27].

**Figure 8:**
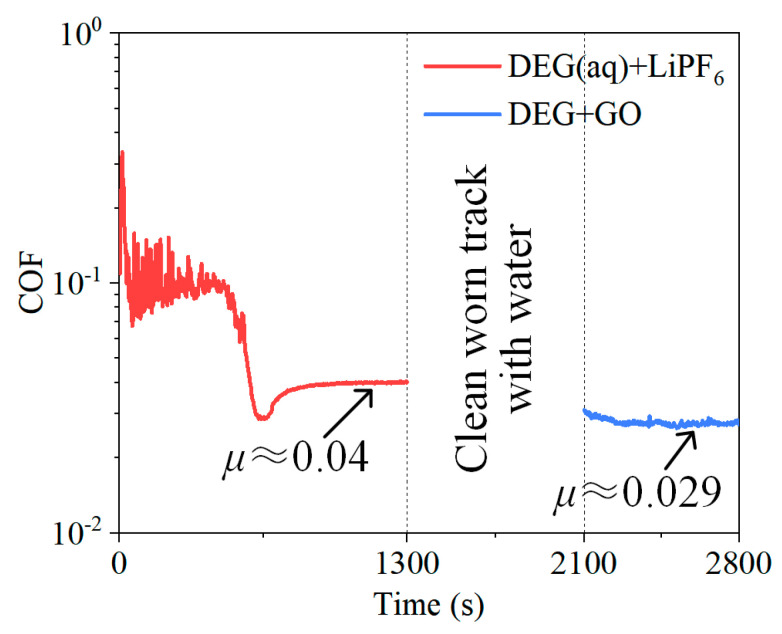
Result to prove GO nanoflakes further reduced COF based on tribochemical layer. Notably, DEG + GO solution contained no water. The conditions were 3 N, 100 mm/s, and 20 μL.

To further study the synergy effect of LiPF_6_ and GO in DEG(aq), the following test was performed. Firstly, a conventional friction test was carried out by using LiPF_6_−containing DEG(aq) as the lubricant, and the test was suspended after the COF tended to be steady with a COF value of 0.04. Then, the worn surfaces were cleaned with deionized water to remove the fluid layer and retain the tribochemical layer of LiPF_6_. Then, GO−containing DEG (without water) was dropped on the worn surface and the test was restarted on the same worn track. The aim of this procedure was to form the GO adsorption layer on the tribochemical layer by physical overlaying. The result showed that the COF was further reduced to 0.029 and kept steady for more than 700 s (Figure 8). The properties and functions of the tribochemical layer and GO adsorption layer formed by physical overlaying were inevitably different from that formed “in situ” by directly employing DEG(aq) + GO + LiPF_6_, which may result in a COF larger than 0.01. Despite these, the result of this test still confirmed that the combination of tribochemical layer and GO adsorption layer can further reduce friction.

**Figure 9:**
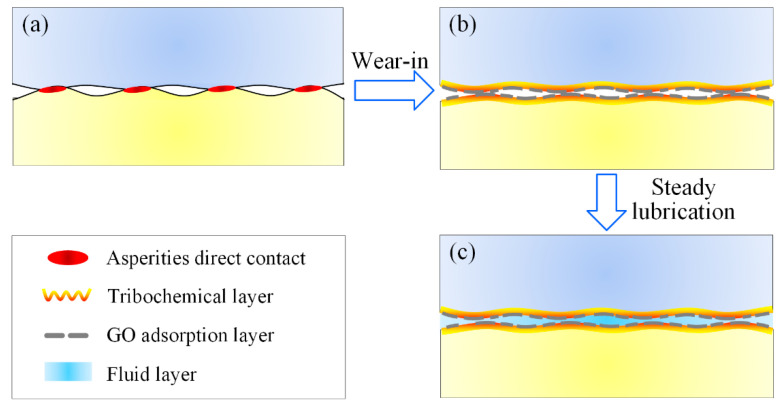
Lubrication model for GO and LiPF_6_ containing DA(aq) solution at macroscale, (**a**) contact between asperities; (**b**) GO adsorption layer and tribochemical layer formed during wear−in stage; (**c**) GO adsorption layer, tribochemical layer, and fluid layer contributed to friction reduction during steady lubrication stage.

According to above analysis, a possible friction model for GO and LiPF_6_ containing DA(aq) was proposed, as shown in Figure 9. The lubrication process contained two stages: the wear−in stage and superlubricity stage. In the wear−in stage, the water molecules in lubricants were volatilized rapidly through the wearing process, during which the lubrication regime was usually located at the boundary lubrication. Hence, the growth in lubricant viscosity caused by water volatilization was conducive to the film formation, which facilitated the improvement in lubrication and led to the drop in COF. Meanwhile, the conflict and wear of solid asperities led to the growth in contact area, and thus the reduction in contact pressure. Therefore, a fluid layer was formed and kept relatively stable at the interface. Moreover, tribochemical reactions induced by rubbing motion led to the formation of a boundary layer containing phosphates, fluorides, and SiO_2_, which resulted in friction reduction as well. In addition to the fluid layer and tribochemical layer, the adhesive effect between contact surfaces and GO led to the adsorption of GO onto the contact surfaces. The GO adsorption layer at the interface could bear a part of the normal load, shield the contact surfaces, and make the shearing interface transfer from solid asperities to GO interlayers; and the weak interlayer interactions of GO further reduce friction during the steady lubrication stage. Therefore, it was the combination of the adsorption layer (GO) and tribochemical layer (LiPF_6_) that significantly contributed to friction reduction in the mixed lubrication regime. Especially for DEG solution, the synergy effect of GO and LiPF_6_ facilitated the realization of superlubricity. Although other factors, such as friction pair materials, high temperature, high speed, heavy load, etc., need to be considered in industrial lubrication, the results of this work proved that lubrication performance can be improved even to the superlubricity level by combining GO with proper materials, and proved GO−family materials as a promising industrial lubricant additive.

## 5. Conclusions

GO and LiPF_6_ were utilized as lubrication additives in DA(aq) solution and apparent friction reduction was achieved at the Si_3_N_4_/sapphire interface. It was concluded that (1) ether bond−containing DA(aq) could achieve superlubricity with the addition of special additives. (2) Despite the viscosity variation of DA, the COF shared the same evolution trend and kept the smallest when both GO and LiPF_6_ were added. (3) The COF during steady stage decreased with decreasing DA viscosity, and superlubricity could be realized and stabilized for nearly 2 h once the viscosity was low enough. (4) For the testing conditions in this study, there were speed range (0.05–0.25 m/s) and load range (1–3 N) for realizing superlubricity. When the sliding speed was lower than 0.05 m/s or the load exceeded 3 N, superlubricity failure would occur. (5) The lubricant volume only affected the duration of wear−in stage and had little effect on COF during the steady lubrication stage. The test and analysis results proved that the synergy effect of GO and LiPF_6_ was the key mechanism for the friction reduction and superlubricity realization. The adsorption layer formed by GO on worn areas bears a part of the load and made the collision of solid asperities transfer to the slip of GO interlayers with extremely low shear stress, to significantly reduce the friction resistance. The tribochemical layer comprised of phosphates and fluorides was formed by LiPF_6_ and contributed to friction reduction. Additionally, the fluid layer also reduced friction resistance. This study proved that, combined with proper materials, GO−family materials as additives could exhibit good lubrication performances and even facilitate the realization of liquid superlubricity at the macroscale.

## Data Availability

Not applicable.
